# Functional Outcomes of Anterior Cruciate Ligament Reconstruction Using Titanium Adjustable Loop Button and Poly-L-co-DL-Lactic Acid-Beta Tricalcium Phosphate (PLDLA-bTCP) Interference Screws: A Single-Center, Retrospective, Observational Study

**DOI:** 10.7759/cureus.34542

**Published:** 2023-02-02

**Authors:** Pulak Sharma, Anurag Baghel, Kumar Keshav, Amit Kumar, Abhishek Singh, Amarendra B Singh

**Affiliations:** 1 Orthopaedics, Sanjay Gandhi Postgraduate Institute of Medical Sciences, Lucknow, IND

**Keywords:** interference screw, adjustable loop, tegner level, lysholm score, anterior cruciate ligament

## Abstract

Background

The anterior cruciate ligament (ACL) reconstruction is a standard surgery in patients with instability of the knee caused by ACL insufficiency. Several differential procedures using grafts and implants such as loops, buttons, and screws have been described. This study aimed to assess the functional outcomes of ACL reconstruction surgery using titanium adjustable loop buttons and poly-L-co-DL-lactic acid-beta tricalcium phosphate (PLDLA-bTCP) interference screws.

Methodology

This was a retrospective, observational, single-center, and clinical study. A total of 42 patients who underwent ACL reconstruction at a tertiary trauma center in northern India between 2018 and 2022 were recruited. Data including demographics, details of the injury, surgery, implants, and surgical outcomes were collected from the patients’ medical records. Further, post-surgery details such as re-injury, adverse events, International Knee Documentation Committee (IKDC) profiles, and Lysholm knee score were recorded from the enrolled patients through a telephonic follow-up. Pain score and Tegner activity scale were used to compare the knee status before and after surgery.

Results

At the time of surgery, the mean age of the recruited patients was 31.1 ± 8.8 years, with a male preponderance of 93%. About 57% of patients had left knee injuries. The common symptoms were instability (67%), pain (62%), swelling (14%), and giving away (5%). During surgery, titanium adjustable loop button and PLDLA-bTCP interference screw implants were used in all patients. The mean follow-up time was 21.2 ± 14.2 months. Based on patient responses, the mean IKDC and Lysholm scores were found to be 54.02 ± 5.93 and 94.4 ± 4.73, respectively. Further, the proportion of patients reporting pain decreased from 62% before surgery to 21% after surgery. The mean Tegner score revealed a significant increase in the activity levels of the patients post-surgery compared to pre-surgery (p < 0.05). Lastly, no adverse events or re-injuries were reported in any of the patients during follow-up.

Conclusions

Our findings revealed a significant improvement in Tegner activity levels and pain scores after surgery. In addition, patient-reported IKDC and Lysholm scores fell under the category of good knee status and function, suggesting a satisfactory functional outcome of ACL reconstruction. Hence, titanium adjustable loop and PLDLA-bTCP interference screws may be a good choice of implants for successful ACL reconstruction surgery.

## Introduction

The anterior cruciate ligament (ACL) is considered one of the most frequently injured ligaments in the human body. ACL functions on a screw-home mechanism, which is imperative for the synchronization of the knee joint to the adjacent joints of the hip and the ankle [1]. The incidence of ACL injury is estimated to be 0.24-0.34 per 1,000 persons per year [2]. Due to its high prevalence, ACL reconstruction surgeries and their outcomes receive considerable attention. About 80-100% of patients reported normal to nearly normal outcome scores after reconstruction [3]. However, approximately 3‑10% of ACL reconstructions fail [4]. The success of ACL reconstruction depends on its ability to heal in the bone tunnels, i.e., tibial and femoral tunnels [5].

Arthroscopically, ACL reconstruction using a hamstring or patella-bone-tendon-bone autograft is the standard surgical treatment for instability [6]. However, the success of this technique critically depends on the ability of the soft-tissue graft to heal inside bone tunnels [7]. To improve the healing and incorporation of the graft, several different graft fixation methods have been devised for ACL reconstruction [8]. Broadly speaking, graft fixation methods are categorized as either aperture fixation (intra-tunnel) or suspensory method (extra-tunnel) [9]. Suspensory fixation devices include fixed (closed)-loop systems and variable (adjustable)-loop systems. Aperture fixation devices include interference screws. Although there is no set pattern of fixation in ACL surgeries, most surgeons prefer a combination of suspensory and aperture fixation [10].

Loop systems and Interference screws are made from various materials, including metals, absorbable ceramics, and inert polymers. Each material has its own advantages and disadvantages. For instance, titanium is considered the most biocompatible metal due to its resistance to corrosion from bodily fluids, bio-inertness, capacity for osseointegration, and high fatigue limit [11]. Thus, a titanium adjustable loop button implant was used for soft-tissue fixation to the bone. Studies in the literature reported that bio-absorbable screws have better shear strength fixation than metallic ones, therefore, interference screws made up of poly-L-co-DL-lactic acid-beta tricalcium phosphate (PLDLA-bTCP) were used for tissue fixation in this study. PLDLA-bTCP is a phosphate ceramic with osteoconductive properties. The composite material is soluble, re-absorbable, maintains pH, exhibits faster degradation, and enhances tissue growth in the affected area due to its porous structure [12].

The right choice of graft fixation devices is one of the most important factors in determining the success of ACL reconstruction and the risk of revision. Due to the scarcity of data on Indian patients on the outcomes and success of a combination of suspensory (titanium adjustable loop button) and aperture (PLDLA-bTCP interference screws) fixation systems in ACL reconstruction surgeries, a retrospective study was undertaken. This study aimed to evaluate the success of titanium adjustable loop button and PLDLA-bTCP interference screws in ACL reconstruction using patient-reported outcome measures, such as improvement in knee pain, Tegner activity level, Lysholm symptom score, and subjective knee function.

## Materials and methods

Study design

A retrospective, observational, single-center, clinical study was conducted to evaluate the functional outcomes of ACL reconstruction using adjustable loop button and PLDLA-bTCP interference screws. The study was conducted at a tertiary trauma center in northern India for patients who underwent arthroscopic ACL reconstruction between October 2018 and May 2022, and the patients were followed up until December 2022.

Ethical approval

The study protocol was approved by the Institutional Ethical Committee (Sanjay Gandhi Postgraduate Institute of Medical Sciences, Lucknow; PGI/428/2022 approved on November 2, 2022). During the telephonic follow-up, each participant provided verbal consent prior to enrollment. The study was conducted in accordance with the International Council for Harmonization of Technical Requirements for Pharmaceuticals for Human Use (ICH), Medical Device Rules 2017, the International Standard ISO 14155:2020 (Clinical Investigation of Medical Devices for Human Patients), and the Declaration of Helsinki.

Study subjects

Inclusion criteria were (1) patients aged ≥18 years who underwent ACL reconstruction surgery with an adjustable loop button and PLDLA-bTCP interference screws at the femoral and tibial ends, respectively; and (2) patients who were willing to give consent for participation in the study.

Patients with pre-existing medical conditions such as chronic inflammatory, metabolic, or collagen vascular diseases were excluded, as well as those with a history of previous surgery or fracture around the affected area.

Surgical procedure

**Figure 1:**
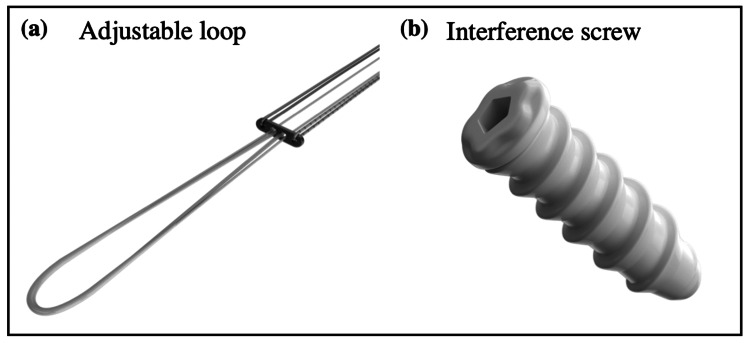
Implants used in the surgical procedure. (a) Titanium adjustable loop and (b) poly-L-co-DL-lactic acid-beta tricalcium phosphate interference screw devices were used in anterior cruciate ligament reconstruction surgery.

All surgical procedures were performed by the same team of experienced surgeons in the hospital. All patients underwent ACL reconstruction using an autologous hamstring graft (semitendinosus and gracilis tendon) from the ipsilateral limb or peroneus longus grafts, or both, along with titanium adjustable loop for femoral fixation and PLDLA-bTCP interference screws for tibial fixation (both implants were from Sironix Arthroscopy Solutions, India) (Figure 1). The meniscal tear, if occurred, was treated with either partial meniscectomy or meniscal repair.

Objectives

The primary objective of the study was to evaluate the functional outcome of ACL reconstruction surgery with titanium adjustable loop button and PLDLA-bTCP interference screws using the International Knee Documentation Committee (IKDC) profile.

The secondary objective was to assess the postoperative knee-specific symptoms, knee activity level, and knee function by recording patient responses to pain scores on the visual analog scale (VAS), Tegner scale, and Lysholm score.

Data collection and outcome measures

The retrospective data including baseline demographics, details of injury, symptoms, surgery details, implants, and surgery protocols were collected from the patient’s medical records. The preoperative clinical evaluation details which consisted of the anterior drawer test (ADT), Lachman test, pivot-shift test, tenderness, and McMurray test were also collected from the records and correlated with MRI findings. Thereafter, the patients were followed up telephonically and data were collected by asking questions using a pre-set questionnaire. The questionnaire was based on patient-reported instruments or outcomes such as pain score, IKDC profile, Tegner activity level, Lysholm scale, any kind of adverse events, postoperative complications, and re-injury. Based on patient responses, the functional outcomes of ACL reconstruction surgery were assessed.

International Knee Documentation Committee Profile

IKDC is a validated scale to assess subjective knee function after ACL reconstruction. It consists of the following three major domains: symptoms such as pain, stiffness, swelling, locking, catching, and giving way; sports activity functions such as stair climbing, squatting, sitting, bending, kneeling, running, and jumping; and the patient’s perception of knee function before and after surgery [13].

Tegner Activity

Tegner knee activity scale is a single-item question to grade daily activity level, work level, or involvement in sports activities, ranging from level 0 (no activity) to 10 (sports at the national level). In the study, the status of the knee was assessed during pre and postoperative periods and compared.

Lysholm Score

The Lysholm knee score, ranging from 0 (worst) to 100 (best), consists of eight questions on pain, instability, locking, swelling, limping, stair climbing, squatting, and the need for support. Based on the patient responses, a score is calculated that indicates surgery outcomes in terms of symptoms and disability. A score of ≥95 indicates no knee problems (excellent), 84-94 indicates problems in sports (good), 65-83 indicates knee problems in sports and sometimes in daily life (fair), and <65 indicates problems in daily life (poor) [14].

Statistical analysis

All statistical analyses were performed using GraphPad software (version 8.0). Descriptive data were reported as mean ± standard deviation, range, or proportions. For continuous variables, the Kolmogorov-Smirnov test was performed to check normality, and then the data were compared using the t-test for normally distributed data. A p-value of ≤0.05 was set for statistically significant data. For adverse events (AEs), the analysis was based on patient counts, not event counts.

## Results

Participants

A total of 42 patients who underwent ACL reconstruction surgery between October 2018 and May 2022 were recruited in the study based on inclusion and exclusion criteria. All these patients were followed-up telephonically and no patients were lost to follow-up. The final follow-up was completed in December 2022.

Demographics and pre-surgery clinical representation

At the time of surgery, the mean age of the recruited patients was 31.1 ± 8.8 years (range = 18-52 years), with 93% of male preponderance (n = 39/42). Among the recruited patients, 57% of patients were full-time working employees (>eight hours), 7% of patients were part-time workers (<five hours), and the remaining 36% of patients were non-workers. The demographic characteristics and clinical representation of the patients are listed in Table 1.

**Table 1:** Demographic characteristics and clinical representation of the patients. n = number of patients; % = percentage; SD = standard deviation

Characteristics, n (%)	Number of patients (n = 42)
Age (years; Mean ± SD)	31.1 ± 8.8
Gender	
- Male	39 (93)
- Female	03 (07)
Employment status	
- Full working (>8 hours)	24 (57)
- Part-time working (<5 hours)	03 (07)
- Not working	15 (36)
Knee injury	
- Left	24 (57)
- Right	18 (43)
Mechanism of injury	
- Sports	38 (90)
- Daily Activities	04 (10)
Meniscal tear	17 (40)
- Medial	12 (29)
- Lateral	04 (10)
- Both	01 (01)
Follow-up time (months; mean ± SD)	21.2 ± 14.2

**Figure 2:**
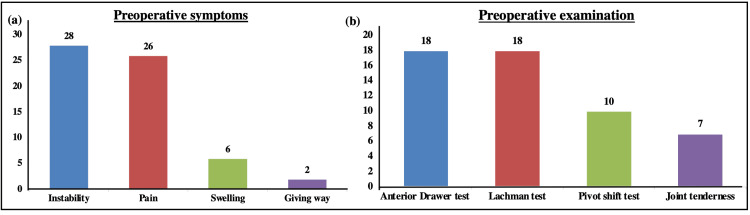
Preoperative presentation of the patients. Bar graphs representing the percentage of patients showing preoperative (a) symptoms and (b) clinical tests.

Clinically, 57% of the patients (n = 24) had left knee injuries, and the remaining 43% of the patients (n = 18) had right knee injuries. Overall, 90% of the injuries occurred due to a fall while participating in recreational sports. Only four patients were injured due to a fall while performing daily activities. About 67% (n = 28/42) of patients presented with instability as a symptom, followed by pain in 62% of patients. Only six patients had swelling, and two patients showed the symptom of giving away (Figure 2a). On examination, 43% (n = 18) of patients tested positive for the ADT and Lachman test, 24% (n = 10) patients for the pivot-shift test, and 17% (n = 7) for joint tenderness, as per the patient medical records (Figure 2b). The time period of injury to the date of surgery ranged from one day to 10 years. A meniscal tear was repaired in 17 (40%) patients. The mean follow-up time was 21.2 ± 14.2 months (range = 7-45 months).

Subjective knee function

**Figure 3:**
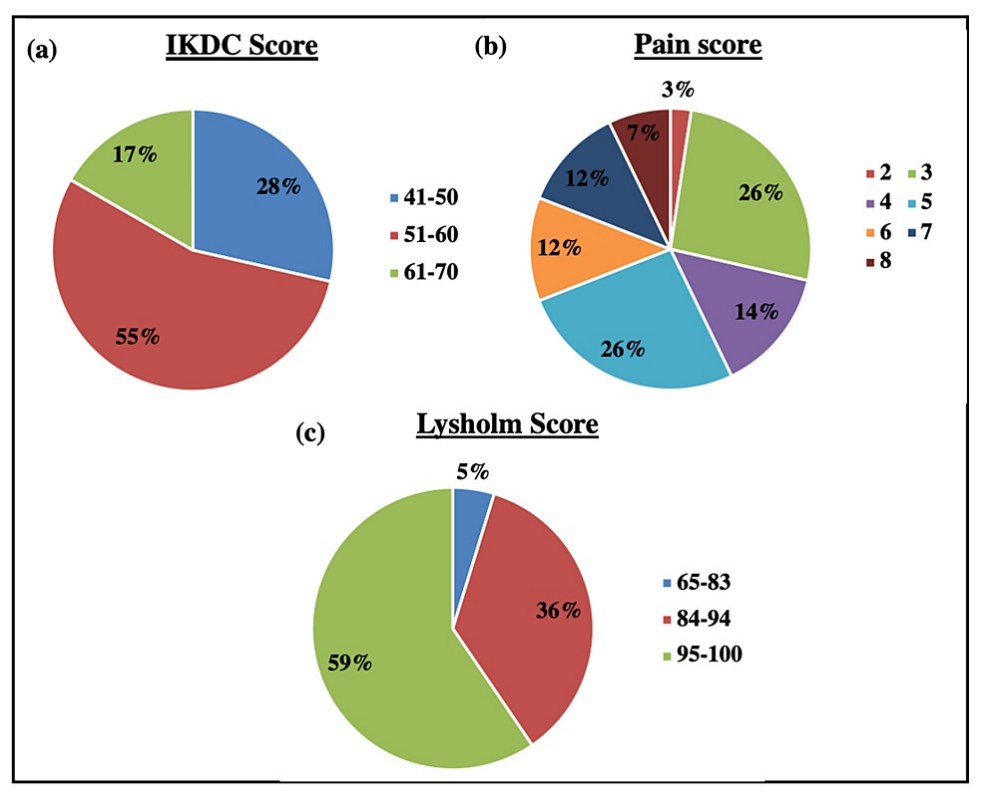
Functional outcomes. Pie charts representing the percentage of patients showing functional outcomes using (a) the IKDC score, (b) the pain score, and (c) the Lysholm score. IKDC = International Knee Documentation Committee

The subjective knee function was assessed postoperatively using the IKDC questionnaire. The mean IKDC score was calculated and found to be 54.02 with a standard deviation of 5.93. About 23 patients had IKDC scoring in the range of 51-60, followed by 12 patients in 41-50, and the remaining seven patients in the 61-70 range (Figure 3, Panel a).

Pain score

Before surgery, 26 (out of 42) patients had reported pain. During the telephonic follow-up, only nine patients reported pain, suggesting a significant decrease in pain after surgery. Further, the patients were asked to score their pain level on VAS, ranging from 0 (no pain) to 10 (worst pain). The proportion of patients’ responses on the pain scale is shown in Figure 3, Panel b. The mean pain score was 4.8 ± 1.6.

Knee-specific symptoms

The knee-specific symptoms were evaluated using the Lysholm score. The postoperative mean score of the patients was found to be 94.4 with a standard deviation of 4.73 (range = 80-100). About 25 patients had excellent Lysholm scores in the range of 95-100, suggesting no knee symptoms. Fifteen patients fell in the category range of 84-94, and two patients were in the fair range (Figure 3, Panel c).

Knee activity

**Figure 4:**
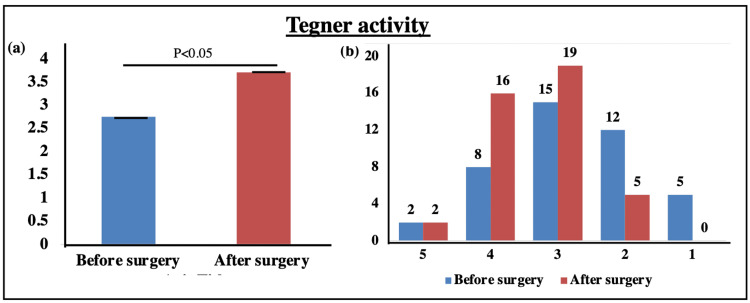
Tegner activity level. Bar graphs representing (a) the mean Tegner activity before and after surgery and (b) the proportion of patients showing Tegner activity levels ranging from 1 to 5.

The knee activity was evaluated using the Tegner scale for both pre and postoperative periods. When pre and postoperative values of Tegner activity levels were compared, a significant increase in the mean score of Tegner level was observed after surgery (2.76 ± 1.05 vs. 3.71 ± 0.55; p < 0.05) (Figure 4a). The Tegner activity levels ranged from 1 to 5 for all recruited patients in the study, which is shown in Figure 4b.

Lastly, no AEs, postoperative complications, or re-injuries were reported by any of the patients during the follow-up.

## Discussion

ACL reconstruction is done to restore the knee’s stability and functionality [15]. The choice of graft and implant fixation options in ACL reconstruction surgery is still evolving. The present study comprised 42 patients who underwent ACL reconstruction surgery and were assessed for functional outcomes using the IKDC, Tegner, and Lysholm scales. All patients were operated on using the Proloop titanium adjustable loop button and Helysis PLDLA-bTCP interference screws in the femoral and tibial ends, respectively. In our study, all recruited patients were aged between 18 and 52 years. Evidence in the literature suggests that age is not a contraindication for patients receiving ACL reconstruction surgery [16,17]. In a study, Brown et al. (2009) reported that females are more prone to ACL injuries [18]. However, the data in our study showed a male preponderance of 93% of patients, possibly because males are more exposed to work in a strenuous environment. In a favored study, Chodavarapu et al. (2017) reported that ACL injury was commonly noted in male patients (96%) [19].

Another similarity was observed in terms of the affected side, i.e., patients with left knee injuries were more common. Notably, the affected side does not influence the outcome of surgery. Based on the medical records of the patients, in our study, 57% of patients had left knee injuries, and the most common symptoms were instability (67%), pain (62%), swelling (14%), and giving away (5%). In the study by Chodavarapu et al. (2017), the author reported 60% of patients with injuries in the left knee, and the most common symptoms were instability (88%), followed by pain (80%) [19]. Our results were consistent with this study.

Several studies have been conducted in the literature to estimate the ideal time between the injury and surgery [19-21]. As noted in our findings also, there is no clear-cut consensus on this because the injury to surgery time ranged from a few weeks to years.

In a study, Kocher et al. (2004) found an association between objective assessment of knee stability and laxity with subjective assessment of symptoms and function [22]. Based on these assessments, the patients were clinically evaluated for ACL reconstruction using the ADT, Lachman test, pivot-shift test, and joint line tenderness test. In our study, positive results for ADT, Lachman test, pivot-shift test, and joint line tenderness test were observed in 43%, 43%, 24%, and 17% of patients, respectively.

Thereafter, the patients were followed up telephonically to review the patient-reported outcomes on pain score, IKDC, Lysholm, and Tegner activity scales. The mean follow-up time was 21.2 ± 14.2 months (range = 7- 45 months). As a part of the primary analysis, the mean IKDC score was calculated and found to be 54.02 ± 5.93. About 55% (n = 23) of patients had IKDC scoring of 51-60, followed by 28% (n = 12) of patients in 41-50, and the remaining 17% (n = 7) of patients in the 61-70 range (Figure 3). Similar mean IKDC scores with the highest percentage of patients in the range of 50-60 were observed in the study by Chodavarapu et al. (2017). Our findings are in concordance with this study [19]. However, the overall mean IKDC score is comparatively lower. The reason being the majority of the patients marked light (walking, housework, or yard work) to moderate physical work (or jogging) as their highest level of activity. The non-involvement of recruited patients in any kind of sports activity or heavy physical work resulted in low IKDC scores.

Further, the postoperative mean Lysholm score of the patients was found to be 94.4 ± 4.73 (range = 80-100), suggesting a good Lysholm score. About 59% of patients had excellent scoring in the range of 95-100, suggesting no knee symptoms, followed by 36% of patients with good scoring (84-94), and 5% with a fair outcome at 65-83 (Figure 3). Remarkably, no patient showed poor Lysholm scores, suggesting good functional outcomes of ACL reconstruction with adjustable loop button and PLDLA-bTCP interference screws. In favor of our data, three studies conducted by William et al. (2004), Bourke et al. (2012), and Chodavarapu et al. (2017) reported a good mean Lysholm score of 90-95 during a follow-up of two years, 15 years and one year, respectively [19,23,24].

The knee activity was evaluated using the Tegner scale for both pre and postoperative periods. The mean scores of pre and postoperative periods were compared and a significant difference in Tegner activity levels was observed (2.76 ± 1.05 vs. 3.71 ± 0.55; p < 0.05) (Figure 4). The Tegner activity levels ranged from 1 to 5 for all patients as the patients belonging to sports activity were very few. In a study, Shervegar et al. (2015) reported that Tegner scores of 3-4 were well-correlated with Lysholm scores in the range of 81-92 [25]. Our findings are in accordance with this study. Lastly, no AEs or re-injuries were reported by any of the patients during the follow-up.

Limitations

There are a few limitations of this study. This was a retrospective study with a telephonic follow-up. No physical examination or such parameters were included in the study. Moreover, no control group was included in the study to compare the functionality of the choice of knee implants as this study focussed on the success of titanium adjustable loop button and PLDLA-bTCP interference screws in ACL reconstruction.

## Conclusions

In recent years, the focus of the healthcare sector has shifted to patients as they are the best evaluators of their health, quality of life, and functional status associated with medicinal products, treatments, services, or therapies. Patients can judge the differences between pre and postoperative periods in terms of pain, stability, safety, and functionality. Hence, patient-reported outcomes are important tools to preview the success of a surgery.

In ACL reconstruction surgeries, Lysholm, IKDC scores, and Tegner activity levels are validated and useful patient-reported outcome measures to analyze the clinical and functional outcomes of the surgery. Our findings revealed a significant improvement in the Tegner activity levels and pain scores after surgery. In addition, patient-reported IKDC and Lysholm scores fall under the category of good knee status and function, suggesting a satisfactory functional outcome of ACL reconstruction. Hence, titanium adjustable loop and PLDLA-bTCP interference screws may be a good choice of implants for successful ACL reconstruction surgery.
